# Antibiotic prescribing behavior among physicians in Asia: a multinational survey

**DOI:** 10.1017/ash.2023.190

**Published:** 2023-06-29

**Authors:** Chonlanan Wiboonchutikula, Hong Bin Kim, Hitoshi Honda, Audrey Yong Xin Loo, Vincent Chi-Chung Cheng, Bernard Camins, Kittiya Jantarathaneewat, Piyaporn Apisarnthanarak, Sasinuch Rutjanawech, Anucha Apisarnthanarak

**Affiliations:** 1 Division of Infectious Diseases, Faculty of Medicine, Thammasat University, Pathum Thani, Thailand; 2 Department of Internal Medicine, Seoul National University Bundang Hospital, Seoul National University College of Medicine, Seongnam, Republic of Korea; 3 Division of Infectious Diseases, Fujita Health University School of Medicine, Toyoake, Aichi, Japan; 4 Division of Pharmacy, Tan Tock Seng Hospital, Singapore; 5 Department of Microbiology, Queen Mary Hospital, Hong Kong Special Administrative Region, China; 6 Division of Infectious Diseases, Department of Medicine, Icahn School of Medicine at Mount Sinai, New York, NY, USA; 7 Research group in Infectious Diseases Epidemiology and Prevention, Faculty of Medicine, Thammasat University, Pathum Thani, Thailand; 8 Center of Excellence in Pharmacy Practice and Management Research, Faculty of Pharmacy, Thammasat University, Pathum Thani, Thailand; 9 Division of Diagnostic Radiology, Department of Radiology, Faculty of Medicine Siriraj Hospital, Mahidol University, Bangkok, Thailand

## Abstract

**Objective:**

To evaluate antibiotic prescribing behavior (APB) among physicians with various specialties in five Asian countries.

**Design:**

Survey of antibiotics prescribing behavior in three stages (initial, on-treatment, and de-escalation stages).

**Methods:**

Participants included internists, infectious diseases (ID) specialists, hematologists, intensivists, and surgeons. Participants’ characteristics, patterns of APB, and perceptions of antimicrobial stewardship were collected. A multivariate analysis was conducted to evaluate factors associated with appropriate APB.

**Results:**

There were 367 participants. The survey response rate was 82.5% (367/445). For the initial stage, different specialties had different choices for empiric treatment. For the on-treatment stage, if the patient does not respond to empiric treatment, most respondents will step up to broader-spectrum antibiotics (273/367: 74.39%). For the de-escalation stage, the rate of de-escalation was 10%–60% depending on the specialty. Most respondents would de-escalate antibiotics based on guidelines (250/367: 68.12%). De-escalation was mostly reported by ID specialists (66/106: 62.26%). Respondents who reported that they performed laboratory investigations prior to empirical antibiotic prescriptions (aOR = 2.83) were associated with appropriate use, while respondents who reported ID consultation were associated with appropriate antibiotic management for infections not responding to empiric treatment (aOR = 40.87); adherence with national guidelines (aOR = 2.57) was associated with reported successful carbapenem de-escalation.

**Conclusion:**

This study highlights the variation in practices and gaps in appropriate APB on three stages of antibiotic prescription among different specialties. Education on appropriate investigation, partnership with ID specialist, and availability and adherence with national guidelines are critical to help guide appropriate APB among different specialties.

## Introduction

Inappropriate antibiotic prescriptions lead to the selection of drug-resistant organisms,^1^ adverse drug reactions,^2^ and increased costs of patient care.^3^ According to Centers for Disease Control and Prevention (CDC), an estimated 50% of antibiotics prescribed in the Unites States is inappropriate.^4^ Antibiotic prescriptions are divided into three stages (initial stage, on-treatment stage, and de-escalation stage).^5^ At the initial stage, physicians often use broad-spectrum antibiotics, even in nonbacterial infections without appropriate investigations. During the on-treatment stage, physicians may not perform appropriate diagnostic tests (cultures of specimens, susceptibility tests) or may not adjust antibiotics according to microbiological culture results. Additionally, during the de-escalation stage, a physician may not recognize the need for de-escalation or discontinuation of antibiotics. Integration of behavior theory to evaluate antibiotic prescribing behaviors (APB) is needed. A previous study suggested that transtheoretical model (TTM) of health behavior change can be used to evaluate APB among physicians.^6^ Physicians who are in higher stages of TTM are assumed to exhibit appropriate APB.

It is well recognized that physicians from various specialties have different APB.^7^ Internists, hematologists, and intensivists often prescribe broad-spectrum antibiotics in febrile neutropenia or in critically ill patients when not indicated, while surgeons prescribe multiple courses of antibiotics and escalate empiric treatment when not indicated.^8–10^ To limit unnecessary antibiotic prescriptions, it is important to understand APB among physicians in various specialties. This information will help inform appropriate intervention to improve appropriate APB for various specialties. We therefore performed a multinational survey to evaluate APB among physicians in various specialties and factors predicting appropriate APB in each stage.

## Methods

The survey was conducted in five countries (Thailand, Singapore, Hong Kong, Korea, and Japan). Participants of each specialty (internist, infectious disease (ID) specialist, hematologist, intensivist, and surgeon) were invited to participate in the survey based on a convenience sample. Prior to the survey, the questionnaire was assessed by all investigators to verify that it covered the information needed to achieve the study goals. The survey was performed from November 1 to December 31, 2022. Google forms were created and distributed through texts (LINE application) or email. Institutional ethical committee approval was obtained prior to the conduct of this survey.

Information collected included age, gender, specialty, level of training, country, total number of beds of the hospital where the participant primarily practiced, availability of ID consultants, and patterns of APB among various specialties. The survey collected data on patterns of antibiotic use, perceptions on the practice of antimicrobial stewardship, adherence to national prescribing guidelines, and analysis of factors associated with prescribing behaviors. It was divided into three stages (initial stage, on-treatment stage, and de-escalation stage). In the initial stage, respondents ranked the top three most common indications for antibiotic prescriptions, chose routine investigations prior to prescribing antibiotics, selected typical empiric antibiotics, reported on influences on prescribing antibiotics, and their antibiotic behavior based on the five stages from the TTM.^11^ TTM stage was categorized into five stages: precontemplation, contemplation, preparation, action, and maintenance. Precontemplation is the first stage in which people do not intend to change their behavior in the upcoming future. The next stage is contemplation; people are starting to be aware of the situation and consider changing their actions. Preparation is the stage in which people decide to act in the near future. Action is the stage in which people begin to change their behavior. The last stage is maintenance (MT); at this stage, the behavior has changed and people are trying to maintain it. In the on-treatment stage, respondents were asked to select their investigations and management in two situations; if their patient’s condition is getting worse and if the organism identified is a multidrug-resistant organism. Lastly, in the de-escalation stage, respondents were asked to choose factors that influence antibiotic de-escalation, frequency of antibiotic de-escalation, and their experience in successful carbapenem de-escalation.

Definitions of appropriate antibiotic prescriptions for each stage were defined as follows. First, in the initial stage, appropriate empirical antibiotics were defined as the administration of proper empirical antibiotics based on the local antibiogram and the adopted institutional guideline for each specific indication.^12–14^ For the on-treatment stage, appropriate management for infections not responding to empiric treatment was defined as appropriate investigations (eg, molecular testing and drug-resistant testing for multidrug resistance together with appropriate management (eg, adjust antibiotic base on antibiogram, remove the source of infection). For the de-escalation stage, successful carbapenem de-escalation was defined as reporting successful carbapenem de-escalation more than 80% in their clinical practice.

All analyses were performed using SPSS, version 26 (Armonk, NY). χ^2^ tests were used to compare categorical variables. Independent t-tests were used for continuous data. All *P* values were two-tailed, and *P* < .05 was considered statistically significant. A multivariate analysis was conducted to evaluate factors associated with certain APB in various specialties in three stages. Adjusted odd ratios (aORs) and 95% confidence intervals (CIs) were calculated.

## Results

Overall, 367 physicians from five countries participated in the survey. The overall survey response rate was 82.47% (367/445). The response rates (responded/invited) were from Thailand (94/100, 94.00%), Korea (92/100, 92.00%), Singapore (61/80, 76.25%), Japan (74/100, 74.00%), and Hong Kong (46/65, 70.77%). The proportions of each participant were from Thailand (94/367, 25.61%), Korea (92/367, 25.07%), Japan (74/367, 20.16%), Singapore (61/367, 16.62%), and Hong Kong (46/367, 12.53%). The middle age range of participants was 31–40 years. The majority of participants were male (217/367, 59.13%). Most participants were staff (235/367: 64.03%) and were from the department of infectious diseases (106/367: 28.88%), internal medicine (91/367: 24.80%), and surgery (64/367: 17.44%). Almost all had ID consultants available at their respective hospitals (337/367, 91.83%). For the behavioral assessment of prescribing practice, most physicians were categorized in the MT stage of TTM (233/367, 63.49%). Characteristics of the study population and APB are summarized in Table 1. The three most common indications for antibiotic prescriptions were pneumonia (n = 155, 42.23%), urinary tract infection (n = 55, 14.99%), and bacteremia (n = 50, 13.62%). The distribution of indications for treatment in various specialties is shown in Table 2.

**Table 1.** Characteristics of survey participants stratified by specialty

Characteristics	Total (N = 367)	Infectious disease specialist (N = 106)	Internist (N = 91)	Surgeon (N = 64)	Intensivist (N = 57)	Hematologist (N = 49)	*P* value^a^
Age range, n (%)							<.001
≤30 years old	73 (19.89)	3 (2.83)	36 (39.56)	19 (29.69)	11 (19.30)	4 (8.16)	
31–40 years old	151 (41.14)	46 (43.4)	36 (39.56)	25 (39.06)	23 (40.35)	21 (42.86)	
41–50 years old	106 (28.88)	41 (38.68)	11 (12.09)	18 (28.13)	18 (31.58)	18 (36.73)	
51–60 years old	32 (8.72)	12 (11.32)	7 (7.69)	2 (3.13)	5 (8.77)	6 (12.24)	
>60 years old	5 (1.36)	4 (3.77)	1 (1.10)	0 (0)	0 (0)	0 (0)	
Male, n (%)	217 (59.13)	64 (60.38)	52 (57.14)	40 (62.50)	35 (61.4)	26 (53.06)	.847
Country, n (%)							.066
Korea	92 (25.07)	22 (20.75)	19 (20.88)	15 (23.44)	19 (25.07)	17 (34.69)	
Thailand	94 (25.61)	21 (19.81)	25 (27.47)	24 (37.5)	13 (22.81)	11 (22.45)	
Japan	74 (20.16)	21 (19.81)	25 (27.47)	12 (18.75)	11 (19.30)	5 (10.2)	
Singapore	61 (16.62)	21 (19.81)	13 (14.29)	9 (14.06)	7 (12.28)	11 (22.45)	
Hong Kong	46 (12.53)	21 (19.81)	9 (9.89)	4 (6.25)	7 (12.28)	5 (10.2)	
Level of training, n (%)							<.001
Intern/Resident	82 (22.34)	3 (2.83)	44 (48.35)	20 (31.25)	5 (8.77)	4 (8.16)	
Fellow	50 (13.62)	17 (16.04)	12 (13.19)	6 (9.38)	11 (19.30)	10 (20.41)	
Staff	235 (64.03)	86 (81.13)	35 (38.46)	38 (59.38)	41 (71.93)	35 (71.43)	
Total number of beds, n (%)							.131
250–500	49 (13.35)	13 (12.26)	14 (15.38)	10 (15.63)	7 (12.28)	5 (10.20)	
501–750	72 (19.62)	16 (15.09)	29 (31.87)	9 (14.06)	7 (12.28)	11 (22.45)	
751–1,200	108 (29.43)	33 (31.13)	21 (23.08)	17 (26.56)	20 (35.09)	17 (34.69)	
>1,200	138 (37.6)	44 (41.51)	27 (29.67)	28 (43.75)	23 (40.35)	16 (32.65)	
Availability of ID consultant, n (%)	337 (91.83)	105 (99.06)	80 (87.91)	52 (81.25)	53 (92.98)	47 (95.92)	.001
TTM, n (%)							<.001
Precontemplation	28 (7.63)	3 (2.83)	7 (7.69)	9 (14.06)	4 (7.02)	5 (10.20)	
Contemplation	36 (9.81)	3 (2.83)	11 (12.09)	13 (20.31)	5 (8.77)	4 (8.16)	
Preparation	38 (10.35)	8 (7.55)	10 (10.99)	10 (15.63)	6 (10.53)	4 (8.16)	
Action	32 (8.72)	1 (0.94)	9 (9.89)	8 (12.50)	4 (7.02)	10 (20.41)	
Maintenance	233 (63.49)	91 (85.85)	54 (59.34)	24 (37.50)	38 (66.67)	26 (53.06)	

Note. ID, infectious disease; TTM, transtheoretical model of health behavior change.

a *P* value represents comparison of characteristics among different specialties.

**Table 2.** Characteristics and pattern of antibiotic prescribing behavior among various specialties at three stages

Stage	Variables	Overall (N = 367)	Infectious disease specialist (N = 106)	Internist (N = 91)	Surgeon (N = 64)	Intensivist (N = 57)	Hematologist (N = 49)
Initial stage	Most common indication	Pneumonia^a^	Pneumonia	Pneumonia	IAI	Pneumonia	FN
		155 (42.2)	54 (50.9)	55 (60.4)	25 (39.1)	51 (89.5)	39 (70.6)
	Influence on choice of antibiotic	International guidelines^b^	International guidelines	International guidelines	International guidelines	International guidelines	International guidelines
		209 (56.9)	70 (66.0)	56 (61.6)	24 (37.5)	33 (57.9)	26 (53.1)
	Three most common empiric antibiotics	BL/BI, 3rd cephalosporin, carbepenem	BL/BI, 3rd cephalosporin, vancomycin	3rd cephalosporin, BL/BI, quinolone	3rd cephalosporin, BL/BI, metronidazole	BL/BI, 3rd cephalosporin, carbepenem	BL/BI, 3rd cephalosporin, carbepenem
On-treatment stage	Course of investigation if patient’s condition worsens	Repeat SW^c^	Repeat SW	Repeat SW	Repeat SW	Order antibiotic susceptibility	Repeat SW, order imaging
		286 (77.9)	89 (84.0)	72 (79.1)	42 (65.6)	50 (87.7)	39 (79.6), 39 (79.6)
	Management strategy if patient’s condition worsens	Step up to broad-spectrum AB^d^	Consider source control	Step up to broad-spectrum AB	Step up to broad-spectrum AB	Step up to broad-spectrum AB	Step up to broad-spectrum AB
		273 (73.4)	86 (81.1)	73 (80.2)	48 (75.0)	46 (80.7)	41 (83.7)
	Course of investigation if patient has MDRO infection	Drug resistance testing^e^	Drug resistance testing	Drug resistance testing	Drug resistance testing	Drug resistance testing	Drug resistance testing
		265 (72.2)	73 (68.9)	63 (69.2)	41 (64.0)	49 (86.0)	39 (79.6)
De-escalation	Reasons for de-escalation	Follow guidelines for organ-specific infection^f^	Follow guidelines for organ-specific infection	Follow guidelines for organ-specific infection	Follow guidelines for organ-specific infection, ID consultation	Follow guidelines for organ-specific infection	Follow guidelines for organ-specific infection
		250 (68.1)	80 (75.5)	62 (68.1)	31 (48.4), 31 (48.4)	42 (73.7)	35 (71.4)
	Reported proportion of carbapenem de-escalation	61%–80%	61%–80%	61%–80%	41%–60%	41%–60%	41%–60%
	Percentage of de-escalation (if susceptibility shows susceptible to narrow spectrum antibiotic)	134 (36.5)	66 (62.3)	31 (34.1)	15 (23.4)	15 (26.3)	5 (10.2)

Note. IAI, intra-abdominal infections; FN, febrile neutropenia; SW, sepsis work up (including blood culture, urine culture, and sputum culture); AB, antibiotics; ID, infectious disease specialists.

a Other common indications (2^nd^ and 3^rd^): urinary tract infection (55/367: 14.99%), bacteremia (50/367: 13.62%).

b Other influence on antibiotic prescriptions (2^nd^ and 3^rd^): hospital antibiotic stewardship program (214/367: 58.3%), ID suggestion (167/367: 45.5%).

c Other investigations (2^nd^ and 3^rd^): advance imaging, eg, CT or MRI (262/367: 71.39%), drug susceptibility from the previous specimen (237/67: 64.6%).

d Other management (2^nd^ and 3^rd^): get rid of source of infection, eg, drainage and debridement (243/367: 66.2%), consult ID (183/367: 49.9%).

e Other investigation (2^nd^ and 3^rd^): inflammatory markers, eg, ESR and CRP (70/367: 19.1%), no further management (65/367: 17.7%).

f Other reasons for de-escalation (2^nd^ and 3^rd^): culture report (197/367: 53.7%), drug susceptibility report (188/367: 51.2%).

For the initial stage, sepsis workup, including blood cultures, urine cultures, and sputum cultures, was the most common routine investigation performed prior to prescribing antibiotics (280/367, 76.29%). Most physicians prescribed a β-lactam/β-lactamase inhibitor as their empiric antibiotic (251/367, 68.39%) and were mostly influenced by international guidelines for treatment of organ-specific infections (209/367, 56.95%). Different specialties have different patterns of empirical antibiotic use (Table 2). There were no differences in the proportion of appropriate antibiotic administrations among participants in MT versus non-MT stages [90/253 (35.57%) vs 163/253 (64.43%); *P* = .197]. The use of biomarkers/rapid diagnostic tests (RDTs) inclusive of erythrocyte sedimentation rate (ESR), C-reactive protein (CRP), and procalcitonin to help with empiric antibiotic choices were more commonly reported in the initial stage (130/367, 35.42%) compared to on-treatment (98/367, 26.70%) and de-escalation stage (63/367, 17.17%).

For the on-treatment stage, if the patient is not responding to empiric treatment, most participants will step up to broader-spectrum antibiotics (273/367: 74.39%) and will repeat the sepsis workup (286/367: 77.93%) together with multi drug resistance testing (265/367,72.21%) to further identify the pathogens as well as their susceptibility profile. The patterns of workups and management of infections not responding empiric treatment among various specialists are summarized in Table 2. There was no difference in the proportion of appropriate antibiotic administration among participants in MT versus non-MT stages [88/256 (34.38%) vs 168/256 (65.63%); *P* = .197].

For the de-escalation stage, the majority of participants reported de-escalating antibiotic regimens based on guidelines for the treatment of organ-specific infections (250/367, 68.12%). De-escalation was mostly reported by ID specialists (66/106, 62.26%) and internists (31/91, 34.07%), while other specialists reported de-escalation much less frequently (Table 2). However, significantly higher proportion of ID specialists reported de-escalation compared to internists (62.26% vs 34.07%; *P* < .001). Successful de-escalation experiences were reported by 61%–80% among internists and ID specialists and 41%–60% among hematologists, intensivists, and surgeons. Notably, most physicians who adhered with national prescribing guidelines were more likely to have reported successful carbapenem de-escalation (aOR = 2.57; 95% CI, 1.26–5.25). Compared to the non-MT stages, participants who were classified in the MT stage were more likely to prescribe appropriate antibiotics in the de-escalation stage [11/61 (18.03%) vs 50/61 (81.97%); *P* = .001]. Characteristics and patterns of APB among various specialties in the three stages are summarized in Table 2.

For the initial stage, reported behaviors associated with appropriate empiric prescriptions included performing proper investigations prior to administration of empiric antibiotics (aOR = 2.83; 95% CI, 1.40–5.69) while being a trainee (aOR = 0.44; 95% CI, 0.21–0.92) and prescribing antibiotics based on a role model (as opposed to guidelines) (aOR = 0.31; 95% CI, 0.11–0.88) were associated with reported inappropriate empiric antibiotic prescriptions. For the on-treatment stage, behaviors associated with appropriate management infections not responding to therapy included consulting ID (aOR = 40.87; 95% CI, 6.38–261.63) and practicing medicine in Japan (aOR = 4.55; 95% CI, 1.13–18.33), while continuing the same antibiotics without investigation (aOR = 0.08; 95% CI, 0.02–0.32) was associated with reporting inappropriate treatment in the on-treatment stage. For the de-escalation stage, behaviors associated with successful carbapenem de-escalation included practicing medicine in Japan (aOR = 3.84; 95% CI, 1.92–7.70) and compliance with national prescribing guidelines (aOR = 2.57; 95% CI, 1.26–5.25), while practicing as intensivists (aOR = 0.29; 95% CI, 0.10–0.87) and hematologists (aOR = 0.08; 95% CI, 0.01–0.62) was associated with reported failure to de-escalate carbapenem therapy. Factors associated with appropriate antibiotic prescriptions in the three stages are summarized in Table 3.

**Table 3:** . Reported factors associated with appropriate antibiotic prescription during the three stages

Variables	aOR	95% CI
**Initial stage: appropriate empiric antibiotics**		
Proper investigation	2.83	1.40–5.69
Intern/resident	0.44	0.21–0.92
Influenced by role model	0.31	0.11–0.88
**On-treatment stage: appropriate management for nonresponsive infections**		
Consult ID	40.87	6.38–261.63
Japan	4.55	1.13–18.33
Continue the same antibiotics without investigation	0.08	0.02–0.32
**De-escalation stage: successful carbapenem de-escalation**		
Japan	3.84	1.92–7.70
Adherence to national prescribing guidelines	2.57	1.26–5.25
Intensivist	0.29	0.10–0.87
Hematologist	0.08	0.01–0.62

Note. ID, Infectious diseases specialist.

## Discussion

There are several notable findings in our study. First, APB varied based on specialties, inclusive of investigations prior to antibiotic administration in both the initial and on-treatment stages, the pattern of antibiotic prescription, and further management of patients with infections not responding to therapy. Notably, the use of biomarkers was more commonly reported in the initial stage, while participants who were in the MT stage of TTM were more likely to prescribe appropriate antibiotics in the de-escalation stage. Second, adequate investigation, inclusive of source control during the on-treatment stage, was only reported by ID specialists. Third, antibiotic de-escalation was commonly reported only by ID specialists, while successful carbapenem de-escalation was reported by 41%–80% of respondents as categorized by specialty. To the best of our knowledge, this study is the first to explore APB among different specialties by a multinational survey in Asia. This knowledge will help identify the gap of antibiotic administration in different stages to help promote appropriate antibiotic prescriptions among physicians in different specialties. Further investigation should be conducted to determine why Japanese physicians are much more accepting of the concept of de-escalation of empiric therapy compared to other Asian nationalities.

APB, including the choice of empiric antibiotics and the use of biomarkers to help with empirical in the initial stage, can vary by specialty.^7,15^ While broad-spectrum antibiotics are usually prescribed for empiric treatment among internists, the use of narrow spectrum antibiotics followed by escalation to broader-spectrum antibiotics are usually observed among surgeons.^16^ Additionally, several studies found that incorrect antibiotic dosing and inappropriate use of broad-spectrum antibiotics such as carbapenems and vancomycin are common practices in treating patients with febrile neutropenia and those in critical care settings.^16,17^ Similarly, our study found that the patterns of empiric antibiotic prescription among specialties can differ based on common sites of infections as well as the adopted international guidelines in each country. Inappropriate APB was commonly reported by trainees, and these young physicians were influenced by their role models suggesting the importance education in younger groups of physicians in all specialties. Several guidelines and consensus suggest the benefit of RDTs and biomarkers, including procalcitonin to help reduce inappropriate antibiotic use during the initial, on-treatment, and de-escalation/cessation stage.^18,19^ These RDTs and biomarkers can be integrated in antibiotic stewardship to improve the diagnostic and therapeutic management for various indications, including acute respiratory infections and sepsis.^20^ However, our study found that use of these RDTs and biomarkers was limited to procalcitonin and mostly only used in the initial stage. It is therefore imperative that more education is provided for the appropriate use of RDTs and biomarkers in all stages.

During the on-treatment stage, adding or switching to broader-spectrum antibiotics is the most common practice if the patients fail to respond to antibiotics therapy.^21^ Failure to respond to antibiotic therapy may occur due to a miss diagnosis, an infectious complication, or the lack of source control.^22^ Our study found that only ID specialists consistently consider source control, while other specialties chose to step up to broader-spectrum antibiotics with/without appropriate investigations. Several studies have shown the benefit of ID consultation to assist with appropriate investigation and antibiotics administration in different clinical sites.^23–25^ ID consultation may include recommending the appropriate choice of antibiotics and reducing the use of broad-spectrum antibiotics, which result in the reduction in antibiotic expenditure as well as improved clinical outcomes.^26^ Likewise, we found that ID consultation was associated with appropriate management for patients who fail to respond during the on-treatment stage. National antibiotic guidelines and policies have been shown to increase awareness of the harm of over prescription of antibiotics and reduce the inappropriate use of antibiotics.^27^ In Japan, implementation a national antibiotic policy was associated with reducing in total antibiotics use.^28^ Our data emphasize the important role of the implementation of national guidelines to help with appropriate management of antibiotics during the on-treatment stage.

Antibiotic de-escalation has been shown to reduce the risk of antimicrobial resistance and adverse effects from unnecessary antibiotic use.^29^ Predictors for antibiotic de-escalation include positive microbiological culture results, consultation with an ID specialist, and adherence to national guidelines.^24,30^ Carbapenem de-escalation has been reported to be 45%–50% by several studies.^31,32^ In our study, the rate of de-escalation was reported to be 10%–60% depending on the specialty. Notably, de-escalation was less commonly reported by hematologists and intensivists, and this may be due to the severity of illness among their patient population. Adherence to national prescribing guidelines has been shown to help with carbapenem de-escalation.^30^ Likewise, our study found that adherence to national prescribing guidelines is associated with appropriate de-escalation. In addition, several studies have reported on the use of behavioral theories to evaluate APB.^33^ A Thai study showed a strong correlation between the TTM stages and appropriate antibiotic prescriptions in surgical wards during the initial stage.^6^ Likewise, our study found that participants who are in the MT stage were more likely to report appropriate APB in the de-escalation stage. Therefore, it is important to provide more education on the appropriate use of APB among participants who belong to the earlier TTM stages during de-escalation.

Our study had several limitations. First, the nature of a self-reported survey lends to a situation in which the actual practice may not reflect what participants report. However, we were very clear to potential participants that the survey is de-identified and completely anonymous and so the reported behavior is more reflective of the practice. Second, the nature of a convenience sample survey may be subject to selection biases. Third, as the majority of the participants were staff, being internists and ID specialists, and were from Korea, Thailand, and Japan, it is possible that our results may skew toward the practices in these specialties and countries. Lastly, the small sample size may limit our ability to identify other factors associated with APB in the initial, on-treatment, and de-escalation stages.

In conclusion, this study shows that there are differences in APB in the initial, on-treatment, and de-escalation stages among different specialties. These differences in behaviors can be reduced by educating prescribers on the use of RDTs and biomarkers during the initial stage, consulting ID specialists during the on-treatment stage, and increased adherence to national antibiotic prescribing guidelines during the de-escalation stage.
